# A Substitute for Cod-Liver Oil in Skin Diseases

**Published:** 1878-05

**Authors:** 


A Substitute for Cod-Liver Oil in Skin Diseases. It is well known that the "cake" which remains after the expression of linseed oil, is largely used by farmers and horse fanciers to fatten their cattle and horses, and to improve the appearance of their coats. This cake contains the principal nutrient albuminoid elements of the ground flaxseed, together with a varying proportion of the oil.

Having had of late a number of cases of cutaneous disease, in which marasmus from defective assimilation of the hydrocarbons was a prominent feature, and in which cod-liver oil was not well borne, it occurred to the writer that the oil of the flaxseed might prove an efficient substitute.

In its ordinary commercial condition, linseed oil is not a very palatable article of diet, but as met with in its natural combination in the fresh seed, is by no means unpleasant to the taste. Believing that the same effects might be expected in the human subject as are known to follow the use of linseed in the lower animals, I have made it a portion of the diet of a number of patients who were unable to take cod-livor oil in the ordinary manner.

The better qualities of flaxseed contain about thirty per cent. of oil, so that by the use of the unpressed seed, a very considerable quantity of oleaginous matter can be incorporated in the daily diet. The seed may be used in several ways: First, the freshly ground seed may be taken in the mouth and thoroughly masticated before swallowing; second, it may be given suspended in milk; and third, the unbroken seed itself may be used. This last method is the one that I prefer. To carry this out, I commonly direct the patient to carry in his pocket or other receptacle a quantity of the seed, and from time to time to take a little of it in his mouth, and to chew it thoroughly before swallowing, in order to insure complete insalivation. In this way some patients will consume several ounces a day, the amount, of course, varying greatly in different cases.

Thus far this use of the seed has not been attended with any disagreeable accompaniments. The stools are rendered easy and natural, without any tendency to diarrhœa, or any other unpleasant complications.

The cases of pemphigus foliaceus, pityriasis rubra, lichen planus, and lichen ruber, which were some time since exhibited at the Society, have been taking the seed in the manner indicated with very decided benefit. It will be remembered that they were all in a more or less marasmic condition when first shown. During the use of the seed, however, they have greatly improved in general nutrition and in the condition of the skin.

The ordinary seed of the drug-stores is not the best that can be obtained for this purpose. A much better article being that known as Calcutta seed. Care should be taken that it is free from admixture with other seeds, chaff, dirt, etc.

As a substitute, in many cases, for cod-liver oil, we believe that it will be found, on further trial, to fully justify our earlier expectations concerning it.

In view of the fact that there is so much sophisticated cod-liver oil in the market, and that an inferior quality can be readily disguised under the form of an "emulsion," a substitute that cannot be readily adulterated would seem to merit the consideration of the profession, and more especially that of dermatologists, in view of what I must consider its specific determination.—*N. Y. Medical Record.*

